# Identification of Hub genes in melasma using integrated transcriptomic analysis

**DOI:** 10.6026/973206300220001

**Published:** 2026-01-31

**Authors:** Akhtar Veg, Mohd Murshad Ahmed, Rafat Ali, Romana Ishrat

**Affiliations:** 1 Centre for Interdisciplinary Research in Basic Sciences (CIRBSc), Jamia Millia Islamia, New Delhi-110025, India; 2 Albert Einstein College of Medicine, Yeshiva University, New York City; 3 Department of Biosciences, Jamia Millia Islamia, New Delhi-110025, India

**Keywords:** Melasma, Hub genes, DEGs, transcriptomic analysis, gene expression profiling

## Abstract

Melasma is a prevalent pigmentary disorder or hyper melanosis skin condition. It is characterized by evenly distributed hyperpigmented regions on the light exposed areas of the facial regions. Its management continues to be a therapeutic challenge due to the limited understanding of their pathogenesis. Therefore, it is of interest to report the molecular mechanism of melasma pathogenesis. By using the identification of key genes and pathways via integrate microarray datasets. These transcriptomics studies showed the complex multifactorial molecular mechanisms are involved in melasma production. These key pathways that are involve in oxidative stress, inflammatory signaling and dermal remodeling. This could give insights into a potential therapeutic target such as DNA repair regulators and metabolic stabilizers.

## Background:

Melasma is an acquired, chronic disorder caused by hormone imbalance, ultraviolet radiation, genetic susceptibility, vascularization, oxidative stress, impaired skin barrier function and others [1, 2]. Melasma affects predominantly women and affects only 10% males [3]. It typically presents as symmetrical hyper melanosis on sun exposed area [4]. This condition affects almost 30% women [5]. Previous transcriptomic studies have found that more than 300 genes exhibiting significant expression differences in lesional vs non lesional skin [6]. High-throughput multi-omics technologies allow deeper investigation of complex molecular interactions [7]. To find the gene expression patterns, microarray technologies have been used extensively [8]. These single gene analyses provide limited information on the collaborative effects of genes within a cell [9]. Biological pathways consist of genes or molecules that work in concert-through chemical reactions, molecular modifications, or signal transduction-to perform these functions [10]. Melasma is an acquired hyperpigmentation which predominantly targets the facial region [11]. It typically presents as chronic, symmetrical facial pigmentation [11]. It negatively impacts patient's overall well-being and directly harms their psychological and emotional health [12]. Although the exact mechanisms remain unclear, it is associated with vascular hyperplasia, chronic inflammation, and impaired barrier function [13]. Lesional skin displays significantly higher levels of the total lipids-including phosphatidic acid, ceramides, and phosphatidylserine- compared with non-lesional skin [14]. The Skin surface lipids functions as barrier for the epidermis which also maintains moisture and elasticity [15]. The changes in number and composition of local lipids have been linked to damage in skin barrier and development of various skin damage [16]. In this study, microarray datasets GSE207744, GSE227015 and GSE185308 were analyzed to explore the molecular mechanisms underlying melasma. Functional analysis using GO, KEGG, and Reactome was performed for differentially expressed genes. Therefore, it is of interest to report hub Genes in Melasma Identified using integrated transcriptomics analysis.

## Methodology:

## Microarray data retrieval:

The R statistical software (Version 4.3.2) was utilized for data downloading and pre-processing [17]. After conducting a comprehensive search, the Gene Expression Omnibus (GEO) dataset accession numbers GSE207744, GSE227015, and GSE185308 were chosen from the National Center for Biotechnology Information (NCBI) [18]. The necessary datasets were downloaded from the Gene Expression Omnibus (GEO) public repository using the GEOquery (Version 2.70.0, R package) [19].

## Data selection criteria:

The database in NCBI was searched for melasma associated microarray data using appropriate keywords: "mRNA expression," "Melasma," and "Homo sapiens" (organism). The following inclusion criteria were applied for the appropriate selection of dataset. (i) Consist of lesional and non-lesional samples, (ii) Specifically containing samples of contrasting pigmentation (lightly and darkly pigmented) and (iii) UV exposure (UV and No-UV). This approach ensured that our analysis encompassed the comprehensive examination of gene expression changes associated with Melasma.

## Patient's characteristics:

Our study included a total of 119 patients from all three selected series summarized in Table 1. The series GSE207744 included 72 patients in which 24 were non lesional while 48 were lesional. The second series GSE227015 included 30 patients out of which 14 were lightly pigmented while 16 were darkly pigmented. The third selected series GSE185308 contains 17 patients in which 12 were exposed to UV and rest of them were not.

## Dataset pre-processing:

Data pre-processing steps, including background correction, normalization, log2 transformation, probe ID mapping, and removal of outlier samples, were performed in for each expression profile downloaded from GEO prior to differential analysis. Probe IDs were mapped to gene symbols using Annotation packages available on Bioconductor [20]. This is common for multiple probes to map to a single gene symbol. These were treated as independent observations in the analysis, and the standardized measures (specifically p-values) were used to summarize the results. The gene symbols corresponding to the smallest p-value for each probe were selected for further analysis. Any unmapped probe gene IDs were excluded from the count matrix.

## Identification of DEGs:

The differential expression analysis (DEGs) of the gene expression profiles from the selected datasets was conducted using the Limma (Version 3.58.1,) and edgeR (Version 4.0.16,) packages in R statistical software [21, 22]. The False Discovery Rate (FDR) method, as outlined by the Benjamini-Hochberg (BH) procedure, was used to adjust the p-values. Differentially expressed genes (DEGs) were recognized based on statistical significance with a threshold P-values < 0.05. Common DEGs across the three selected datasets were then selected for further analysis.

## Gene ontology and KEGG-pathway enrichment analysis:

Biological significance of DEGs was explored by Gene Ontology Enrichment Analysis including biological process, cellular component and molecular function. The KEGG pathway enrichment analysis (KEGG, REACTOME, WikiPathways) were performed based on gprofiler2 (Version 0.2.3,) packages by applying a significance threshold of FDR-corrected p-values (< 0.05) [23].

## Protein-Protein Interaction (PPI) network analysis and hub gene analysis:

All human PPIs were retrieved from the STRING database (Version 12.0,) using the StringApp plugin (Version 2.0.3,) in Cytoscape (Version 3.10.2) [24,25-26]. To identify and analyze key central regulatory genes within the PPI network, we conducted a module analysis using the CytoHubba application (Version 0.1,) with default parameters in Cytoscape [27]. Various topological algorithms, including Degree, Maximal Clique Centrality (MCC), Maximum Neighborhood component (MNC), and Edge Percolated Component (EPC). Also, the network centrality measures such as Betweenness, Bottleneck, Stress, Radiality, and Closeness, were employed through the CytoHubba plugin to construct networks for the top 20 nodes.

## Results and Discussion:

**Figure 1:**
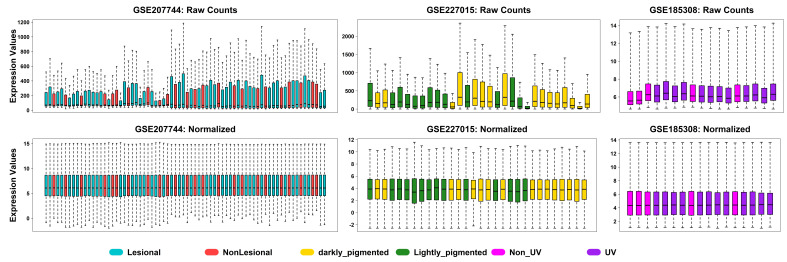
Boxplot showing the distribution of gene expression values across all samples after normalization.

The GEO repository provided the datasets GSE207744, GSE227015, and GSE185308, which were then, processed using conventional normalization pipelines. The reduction of technical variability was guaranteed by TMM normalization for RNA datasets and quantile normalization for microarray platforms. The quality of the processed datasets was validated by boxplots of the normalized data (Figure 1), which showed a consistent distribution of expression values across samples. In transcriptomic analysis, proper normalization is crucial because it preserves the accuracy of subsequent statistical interpretation.

**Figure 3:**
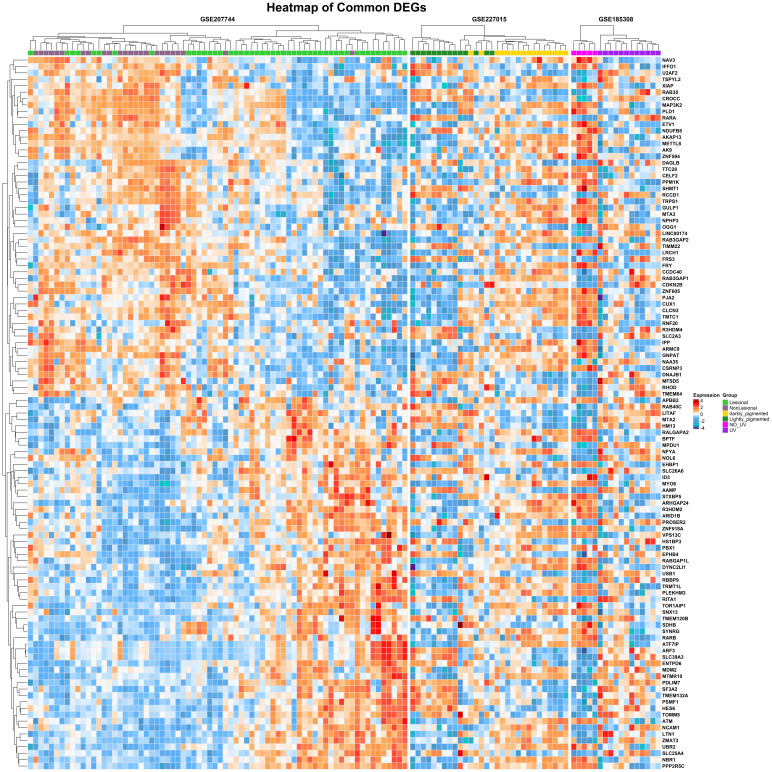
Heat map of 111 common DEGs across all datasets. Samples are clustered based on gene expression profiles, highlighting consistent differential expression across multiple conditions.

**Figure 4:**
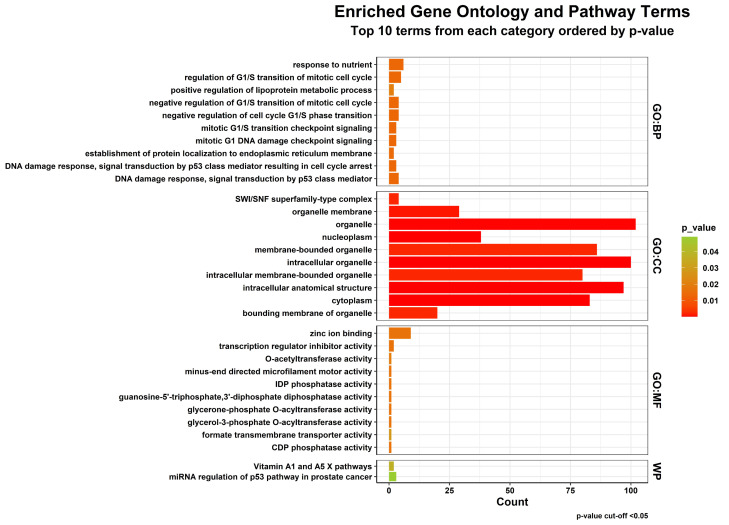
Gene Ontology enrichment results for the common DEGs. Bar plots show the top significantly enriched biological processes, molecular functions, and cellular components.

**Figure 6:**
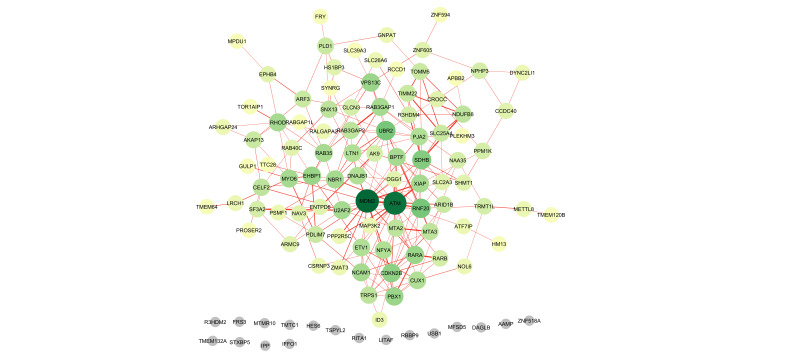
Protein-protein interaction (PPI) network of the 111 common DEGs constructed using the STRING database and visualized in Cystoscape.

**Figure 7:**
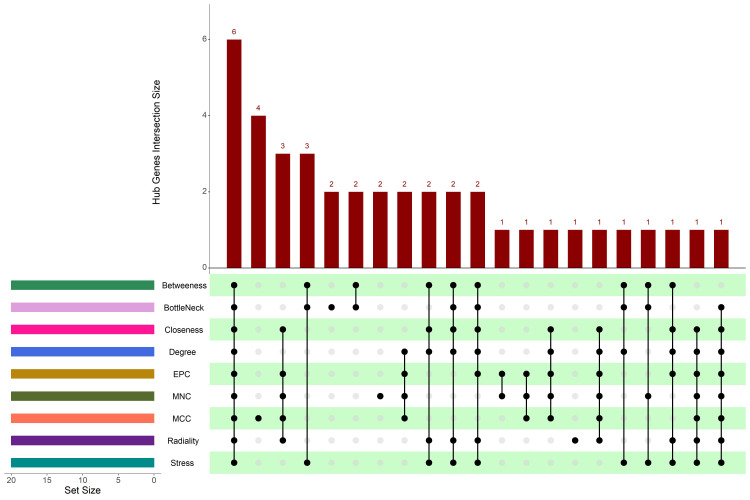
UpSet plot illustrating the overlap of top hub genes identified across nine different CytoHubba algorithms. The intersection highlights six consistently ranked hub genes: MDM2, ATM, SDHB, UBR2, RNF20, and PJA2.

The Figure 1a showed the raw and normalized gene distribution from series GSE207744. The Figure 1b and Figure 1c also showed the raw and normalized gene distribution from series GSE227015 and GSE185308 respectively. Differential expression analysis revealed 4,963 genes between samples with light and dark pigmentation, 3,563 genes between lesional and non-lesional skin, and 3,073 genes between samples exposed to UV light and those that were not. The widespread dysregulation that takes place in these circumstances is reflected in the volcano plots for each dataset (Figure 2a, Figure 2b- Figure 2c). When all datasets were integrated, 111 genes were consistently dysregulated. Strong clustering of disease-associated signatures independent of sample origin is demonstrated by the expression patterns of these common DEGs in the heatmap (Figure 3), demonstrating their biological significance. Pathways linked to oxidative stress, inflammation, mitochondrial metabolism, DNA damage response, and ubiquitin-mediated protein regulation were significantly overrepresented. This is described through functional enrichment analyses using GO, KEGG, Reactome, and WikiPathways. Gene Ontology enrichment (Figure 4) demonstrated the role of processes associated with cell cycle regulation, protein modification, and cellular stress responses. Lesional, pigmented, and UV-exposed groups were clearly segregated by PCA analysis (Figure 5a, Figure 5b, Figure 5c, Figure 5d), confirming different expression signatures. The 111 common DEGs showed significant connectivity in a protein-protein interaction (PPI) network created with STRING and displayed in Cytoscape (Figure 6). We identified the top 10 genes from the PPI network based on rankings derived from nine different CytoHubba algorithms. These including Betweenness, Bottleneck, Closeness, Degree, Maximum Clique Centrality (MCC), MNC, EPC, Radiality, and Stress. The results of these algorithms for hub gene identification are presented. Six central hub genes were found by topological analysis using nine CytoHubba algorithms: MDM2, ATM, SDHB, UBR2, RNF20, and PJA2. These six genes were verified by UpSet analysis (Figure 7) as the consistently ranked regulators using all techniques. The existence of ATM and MDM2 suggests that the p53-regulated DNA damage response pathway, which is triggered by prolonged UV exposure, plays a key role [28]. In line with earlier findings on melanin pathway upregulation in melasma, persistent activation of this regulatory axis has been linked to melanocyte overstimulation.

The discovery of SDHB supports the theory that oxidative stress plays a major role in melasma pathology by implicating mitochondrial dysfunction and increased production of reactive oxygen species (ROS) [29, 30]. Melanocyte hyperactivity and cellular aging are known to be accelerated by mitochondrial impairment. The ubiquitin-associated genes UBR2, RNF20, and PJA2 also show changes in chromatin regulation and proteostasis, which affect dermal remodeling, keratinocyte-melanocyte communication, and inflammatory signaling [31, 32]. These transcriptomic results are consistent with earlier proteomic and metabolomic data that demonstrate elevated expression of pigmentation- related proteins, Wnt pathway inhibitors (WIF1, SFRP2), inflammatory mediators (VEGF, bFGF) and lipid metabolism proteins linked to dysfunction of the epidermal barrier [11, 33]. Increased prostaglandins and metabolites derived from tryptophan also point to a metabolic imbalance and neuroendocrine involvement. Rather than a single increase in melanogenesis, the evidence points to a multifactorial model of melasma that includes oxidative stress, DNA damage signaling, mitochondrial disruption, inflammation, and impaired tissue remodeling [1]. The chronic and recurrent nature of melasma is explained by the convergence of several biological disorders, which also highlights the need for treatment approaches that go beyond blocking melanin synthesis. The hub genes found here, especially ATM, MDM2, and SDHB, are promising molecular markers for potential therapeutic targeting and diagnostic assessment. Larger sample sizes and functional experiments should be included in future research to confirm these results and make it easier to translate them into clinical recommendations for better melasma treatment.

## Conclusion:

The molecular mechanisms underlying the condition by identifying important differentially expressed genes and proteins involved in melisma is shown using on integrated transcriptomics and proteomic analysis. Potential therapeutic targets and potential biomarkers for diagnosis and treatment are highlighted by the hub genes and protein interactions that have been found. Future research with bigger cohorts and more extensive multi-omics integration is required to validate these targets and create more potent treatment approaches.

## Advancement to knowledge:

Melasma is a common pigment disorder or hyper melaninization dermatosis, which has evenly distributed hyper-pigmented areas in the light-exposed regions of the facial areas. The management of melasma has remained a therapeutic dilemma due to a lack of understanding of the pathogenesis. It, therefore, has great relevance in reporting the mechanism of pathogenesis of melasma at a molecular level through identification of important genes, pathways. This has been achieved through integrated microarray data. The pathogenesis of melasma is complex, as transcriptional and genetic studies indicate involvement of oxidative stress, inflammatory pathways, and dermal reconstruction mechanisms. These pathways may provide potential targets for therapeutic intervention, including regulation of DNA repair mechanisms or metabolism.

## Figures and Tables

**Figure 2a:**
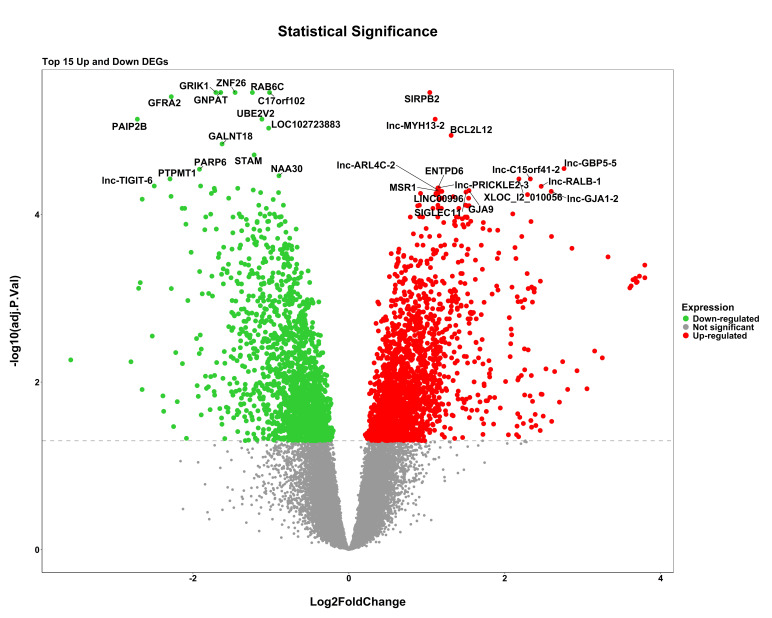
Volcano plots illustrating the distribution of upregulated and downregulated DEGs for GSE207744 dataset comparing lesional vs non-lesional. Red and green dots represent statistically significant upregulated and downregulated genes, respectively.

**Figure 2b:**
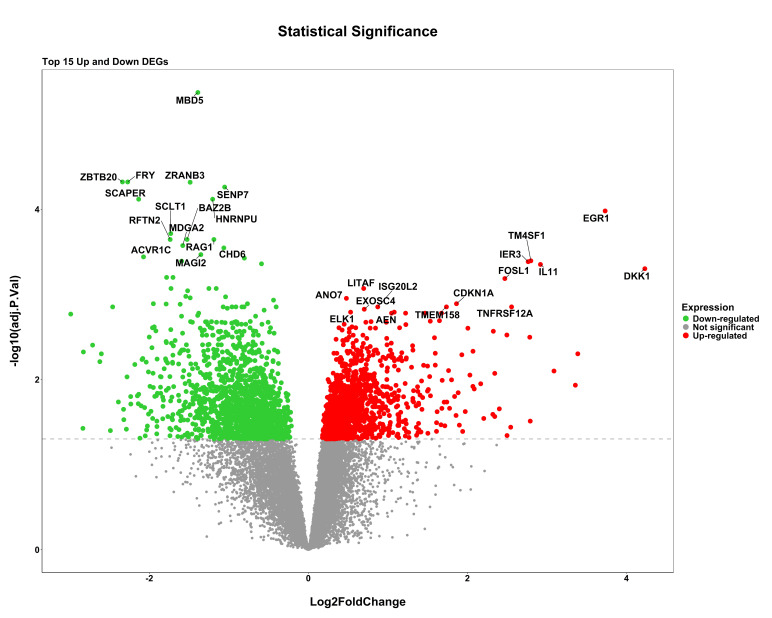
Volcano plots illustrating the distribution of upregulated and downregulated DEGs for GSE227015 dataset comparing lightly pigmented vs darkly pigmented. Red and green dots represent statistically significant upregulated and downregulated genes, respectively.

**Figure 2c:**
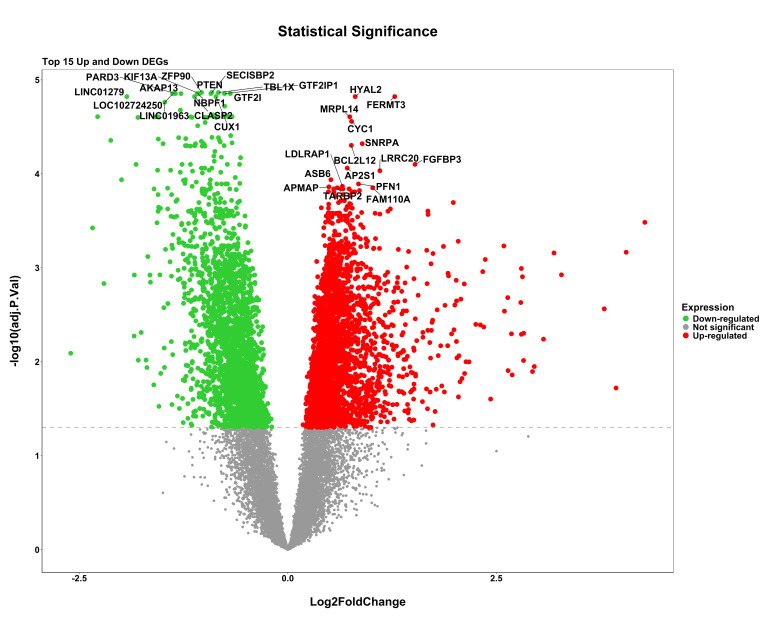
Volcano plots illustrating the distribution of upregulated and downregulated DEGs for GSE185308 dataset comparing UV vs non-UV. Red and green dots represent statistically significant upregulated and downregulated genes, respectively.

**Figure 5a:**
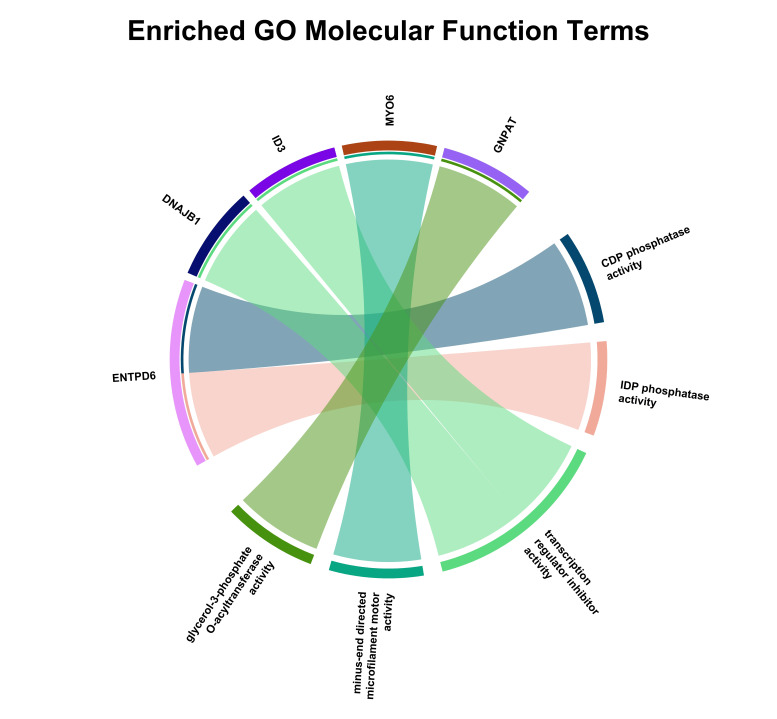
The figure showed the Enriched GO Molecular Function terms. The chord diagram displays significantly enriched molecular function categories in the analyzed gene dataset, with segment size indicating relative enrichment. Inner ribbons denote shared genes among GO terms, highlighting functional overlap between enzymatic and regulatory activities.

**Figure 5b:**
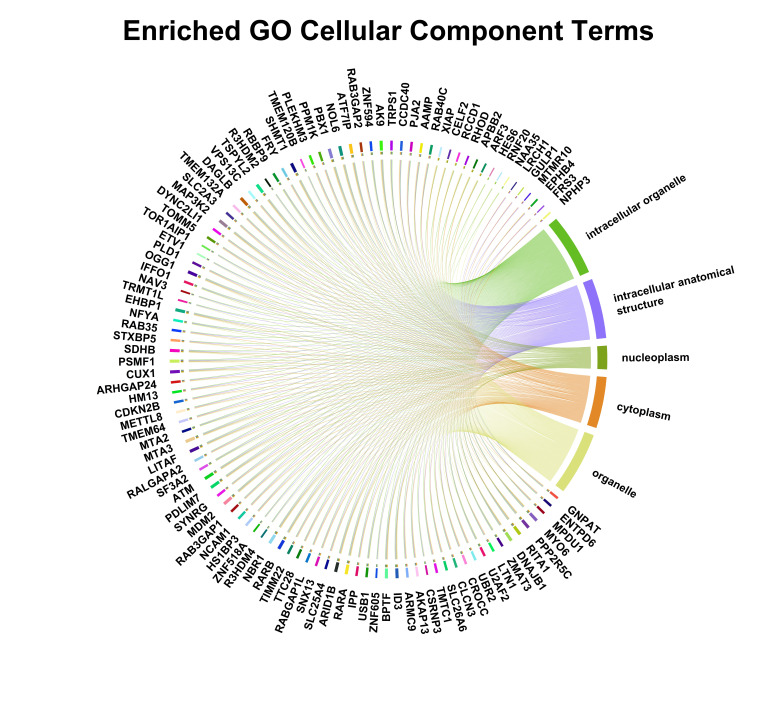
The figure showed Enriched GO Cellular Component terms. The chord diagram depicts significantly enriched GO cellular component categories associated with the analyzed genes. Outer labels represent individual genes and cellular components, while ribbon connections indicate gene-component associations, highlighting predominant localization to intracellular organelles, nucleoplasm, cytoplasm, and related anatomical structures.

**Figure 5c:**
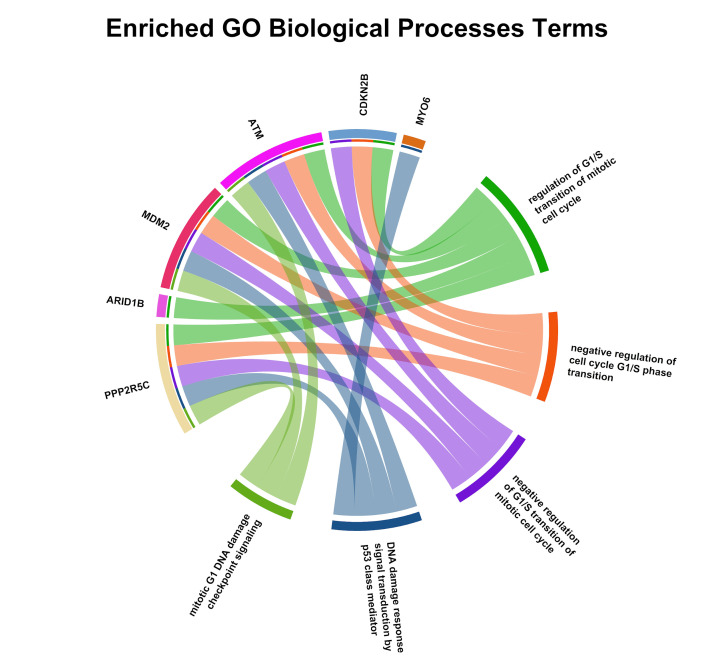
This figure showed Enriched GO Biological Process terms. The chord diagram summarizes significantly enriched GO biological processes linked to the analyzed genes. Segment size reflects the relative contribution of each process, while connecting ribbons indicate shared genes, highlighting enrichment in cell cycle regulation, DNA damage response, mitotic progression, and transcriptional control.

**Figure 5d:**
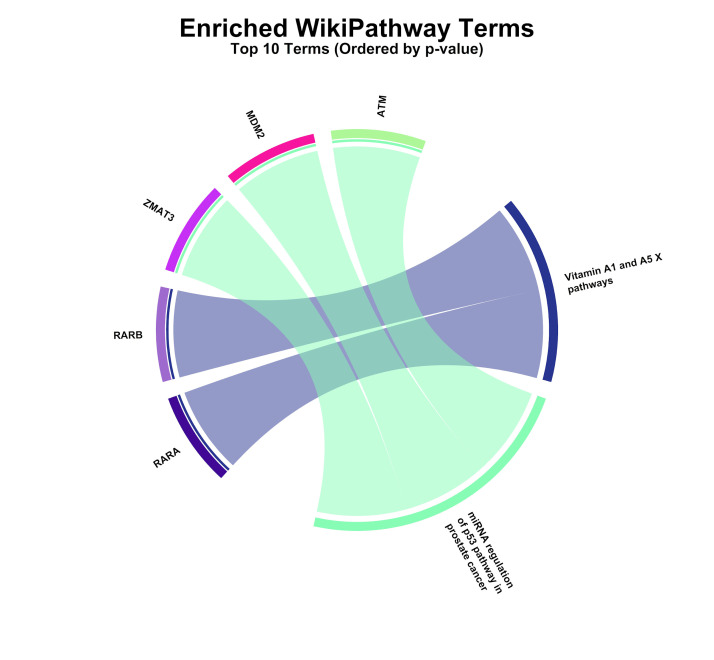
This figure showed Enriched WikiPathway terms. The chord diagram shows the top enriched WikiPathways pathways ranked by p-value for the analyzed gene dataset. Outer segments represent pathways and associated genes, while ribbon connections indicate gene-pathway relationships, highlighting enrichment in vitamin A/retinoic acid signaling and DNA damage response-related pathways.

**Table 1:** The total no of samples, traits, country, year and illness of each downloaded series

**Series**	**Sample**	**traits**	**Total sample**	**Country**	**year**	**Illness**
	24	Non Lesional				
GSE207744	48	Lesional	72	France	2022	Melasma
	14	Lightly pigmented				
GSE227015	16	Darkly pigmented	30	USA	2023	Melasma
	12	UV				
GSE185308	5	Non_UV	17	Japan	2022	Melasma
